# A monoclinic polymorph of 4,4′-dichloro-2,2′-(piperazine-1,4-diyl­dimethyl­ene)diphenol

**DOI:** 10.1107/S1600536808038750

**Published:** 2008-11-26

**Authors:** Koji Kubono, Yuki Tsuno, Keita Tani, Kunihiko Yokoi

**Affiliations:** a Division of Natural Sciences, Osaka Kyoiku University, Kashiwara, Osaka 582-8582, Japan

## Abstract

The titile compound, C_18_H_20_Cl_2_N_2_O_2_, crystallizes as a monoclinic form in the space group *P*2_1_/*n*, with *Z′* = 1/2. It is polymorphic with the previously reported orthorhombic form [Kubono, Tsuno, Tani & Yokoi (2008). *Acta Cryst.* E**64**, o2309]. In the present polymorph, the mol­ecule lies on a crystallographic inversion centre at the piperazine ring centroid. An intra­molecular O—H⋯N hydrogen bond forms an *S*(6) ring motif. Inter­molecular C—H⋯O hydrogen bonding generates a *C*(5) chain motif propagating along the *b* axis, forming sheets parallel to (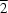
02) with a first-level graph set *S*(6)*C*(5)*R*_6_^6^(34).

## Related literature

For the *Pbca* polymorph, see: Kubono *et al.* (2008 ▶). For graph-set notation in hydrogen bonds, see: Bernstein *et al.* (1995 ▶). For the synthesis of a ligand with two piperazine arms, see: Bharathi *et al.* (2006 ▶). For the monoclinic and ortho­rhom­bic polymorphs of a tetra­chloro-2,2′-(piperazine-1,4-diyl­dimeth­yl­­ene)diphenol, see: Kubono & Yokoi (2007 ▶). For the structure of 1,4-bis­(2-hydr­oxy-5-methyl­benz­yl)piperazine, see: Kuppayee *et al.* (1999 ▶).
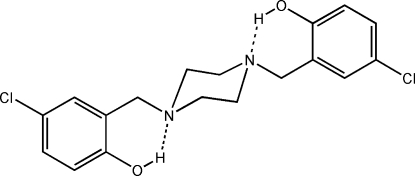


## Experimental

### 

#### Crystal data

C_18_H_20_Cl_2_N_2_O_2_
*M* *_r_* = 367.26
Monoclinic, 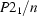

*a* = 15.755 (4) Å
*b* = 9.2667 (17) Å
*c* = 5.9771 (19) Å
β = 96.45 (2)°
*V* = 867.1 (4) Å^3^
*Z* = 2
Mo *K*α radiation
μ = 0.39 mm^−1^
*T* = 298.1 K
0.35 × 0.15 × 0.15 mm

#### Data collection

Rigaku AFC-7R diffractometer
Absorption correction: none
2462 measured reflections
1994 independent reflections
1316 reflections with *F* ^2^ > 2σ(*F* ^2^)
*R*_int_ = 0.020
3 standard reflections every 150 reflections intensity decay: 0.3%

#### Refinement

*R*[*F* ^2^ > 2σ(*F* ^2^)] = 0.041
*wR*(*F* ^2^) = 0.149
*S* = 1.01
1994 reflections
119 parameters
All H-atom parameters refined
Δρ_max_ = 0.54 e Å^−3^
Δρ_min_ = −0.52 e Å^−3^

### 

Data collection: *WinAFC* (Rigaku/MSC, 2006 ▶); cell refinement: *WinAFC*; data reduction: *CrystalStructure* (Rigaku/MSC, 2006 ▶); program(s) used to solve structure: *SIR92* (Altomare *et al.*, 1993 ▶); program(s) used to refine structure: *CRYSTALS* (Betteridge *et al.*, 2003 ▶); molecular graphics: *PLATON* (Spek, 2003 ▶); software used to prepare material for publication: *CrystalStructure*.

## Supplementary Material

Crystal structure: contains datablocks global, I. DOI: 10.1107/S1600536808038750/si2131sup1.cif

Structure factors: contains datablocks II. DOI: 10.1107/S1600536808038750/si2131IIsup2.hkl

Additional supplementary materials:  crystallographic information; 3D view; checkCIF report

## Figures and Tables

**Table 1:** Hydrogen-bond geometry (Å, °)

*D*—H⋯*A*	*D*—H	H⋯*A*	*D*⋯*A*	*D*—H⋯*A*
O1—H1⋯N1	0.83	1.85	2.604 (2)	150
C3—H3⋯O1^i^	0.95	2.60	3.547 (2)	175

Symmetry code: (i) 
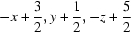
.

**Fig. 1.**
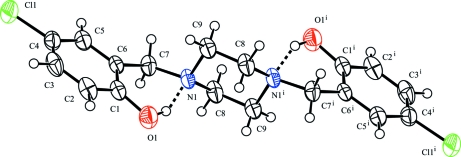
The molecular structure of (II) with the atom-labelling scheme and displacement ellipsoids are drawn at the 50% probability level. H atoms are represented by circles of arbitrary size. [Symmetry code: (i) 1 - x, -y, 2 - z.]

**Fig. 2.**
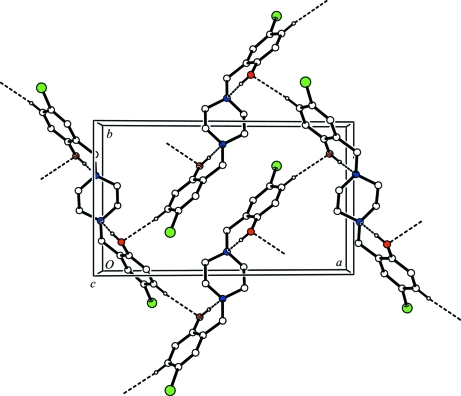
The molecular packing of (II), showing the formation of a sheet with a first level graph set S(6)C(5)R66(34). The hydrogen bonds are shown as dashed lines. The H atoms not involved in the hydrogen bonds have been omitted for clarity.

## supplementary crystallographic information

### Comment

We have recently reported the crystal structure of 4,4'-dichloro-2,2'-(piperazine-1,4-diyldimethylene)diphenol, (I) (Kubono *et al.*, 2008), which crystallizes as an *orthorhombic* form in space group *Pbca* with *Z'* = 1. (I) can act as complexing reagents (Bharathi *et al.*, 2006), so trying to synthesize the zinc complex with this ligand. The crystalline metal complexes were not given, however, a small amount of a new polymorph of the title compound, (II), was obtained from the reaction solution. We report here the molecular and crystal structure of (II).

The molecule of (II) crystallizes in the centrosymmetric space group *P2_1_/n* with *Z'* = 1/2 in the asymmetric unit. The molecular structure of (II) is shown in Fig. 1. The molecule lies on a crystallographic inversion centre at the piperazine ring centroid. It is interesting to note that in the *orthorhombic* form (I) (Kubono *et al.*, 2008) the molecule has a pseudo-inversion centre. The piperazine ring adopts a chair conformation. The bond lengths and angles in (II) are normal and comparable with those in the *orthorhombic* form (I) (Kubono *et al.*, 2008), in the *monoclinic* and *orthorhombic* polymorph of dichlorophenol derivative (Kubono & Yokoi, 2007) and in the *p*-cresol one (Kuppayee *et al.*, 1999). The molecular structures of (I) and (II) are closely similar, so the only slight differences were observed. The maximum differences of bond distance and angle between the two polymorphs are less than 0.02 Å [C1—C2: (I) 1.377 (4) Å, (II) 1.395 (2) Å] and less than 2 ° [C7—N1—C8: (I) 110.6 (2) °, (II) 112.58 (15) °], respectively. The most obvious differences are the torsion angles C5—C6—C7—N1 [(I) 149.3 (2) °, (II) 157.10 (17) °]. The intramolecular O1—H1···N1 hydrogen bond distance is 2.604 (2) Å (Table 1), forming a *S*(6) ring motif (Bernstein *et al.*, 1995).

In the crystal structure of (II), there is intermolecular C—H···O hydrogen bond, involving a aromatic H atom (Table 1). Atom C3 in the molecule at (*x*, *y*, *z*) acts as hydrogen bond donor to atom O1 in the molecule at (3/2 - *x*,1/2 + *y*,5/2 - *z*), so forming a *C*(5) chain running parallel to the [010] direction and generated by the *n*-glide plane at *y* = 1/4. The molecules are linked by the combination of the *S*(6) ring and the *C*(5) chain into a sheet parallel to (202) with a first level graph set *S*(6)*C*(5)*R*_6_^6^(34) (Fig. 2). In the crystal structure of (I), intermolecular C—H···O hydrogen bonds involving methylene H atoms generate *C*(5) chain motifs to form a sheet with a first level graph set *S*(6)*C*(5)*R*_6_^6^(26) (Kubono *et al.*, 2008). Each polymorph is characterized by the hydrogen bonding network structure.

### Experimental

The title compound was prepared by the reaction of 4-chlorophenol, piperazine and paraformaldehyde as described previously (Kubono *et al.* 2008). The *orthorhombic* form, (I) (36.7 mg, 0.10 mmol) was dissolved in 25 ml hot chloroform. Then 15 ml of a methanol solution of zinc acetate dihydrate (22.0 mg, 0.10 mmol) were added to this solution. The mixture was stirred for 20 min at 340 K. After a few days at room temperature, trace amount of column crystals of (II) were picked out manually in the reaction solution. Melting point: 512–514 K. ESI-Ms: *m*/*z*, 367 for [*M*]^+^.

### Refinement

The H atoms of the hydroxyl groups were found from a difference Fourier map. The other H atoms were placed at idealized positions with C—H = 0.95 Å. All the H atoms were refined as a riding model with *U*_iso_(H) = 1.2 *U*_eq_(C).

### Crystal data

**Table:**

C_18_H_20_Cl_2_N_2_O_2_	*F*_000_ = 384.00
*M**_r_* = 367.26	*D*_x_ = 1.407 Mg m^−^^3^
Monoclinic, *P*2_1_/*n*	Mo *K*α radiation λ = 0.71069 Å
Hall symbol: -P 2yn	Cell parameters from 25 reflections
*a* = 15.755 (4) Å	θ = 15.2–17.3º
*b* = 9.2667 (17) Å	µ = 0.39 mm^−^^1^
*c* = 5.9771 (19) Å	*T* = 298.1 K
β = 96.45 (2)º	Column, colorless
*V* = 867.1 (4) Å^3^	0.35 × 0.15 × 0.15 mm
*Z* = 2	

### Data collection

**Table:**

Rigaku AFC-7R diffractometer	θ_max_ = 27.5º
ω–2θ scans	*h* = −20→20
Absorption correction: none	*k* = 0→12
2462 measured reflections	*l* = −7→4
1994 independent reflections	3 standard reflections
1316 reflections with *F*^2^ > 2σ(*F*^2^)	every 150 reflections
*R*_int_ = 0.020	intensity decay: 0.4%

### Refinement

**Table:**

Refinement on *F*^2^	All H-atom parameters refined
*R*[*F*^2^ > 2σ(*F*^2^)] = 0.041	*w* = 1/[0.002*F*_o_^2^ + σ(*F*_o_^2^)]/(4*F*_o_^2^)
*wR*(*F*^2^) = 0.149	(Δ/σ)_max_ < 0.001
*S* = 1.01	Δρ_max_ = 0.54 e Å^−^^3^
1994 reflections	Δρ_min_ = −0.52 e Å^−^^3^
119 parameters	Extinction correction: none

### Special details

**Table:**

Refinement. Refinement was performed using all reflections. The weighted *R*-factor (*wR*) and goodness of fit (*S*) are based on *F*^2^. *R*-factor (gt) are based on *F*. The threshold expression of *F*^2^ > 2.0 σ(*F*^2^) is used only for calculating *R*-factor (gt).

### Fractional atomic coordinates and isotropic or equivalent isotropic displacement parameters (Å^2^)

**Table:**

	*x*	*y*	*z*	*U*_iso_*/*U*_eq_	
Cl1	0.70334 (4)	0.71539 (6)	0.65673 (12)	0.0627 (2)	
O1	0.59890 (10)	0.28682 (15)	1.2938 (2)	0.0497 (4)	
N1	0.50843 (9)	0.15177 (16)	0.9674 (2)	0.0354 (3)	
C1	0.62416 (12)	0.3779 (2)	1.1357 (3)	0.0378 (4)	
C2	0.69309 (12)	0.4691 (2)	1.1990 (3)	0.0461 (5)	
C3	0.71835 (13)	0.5701 (2)	1.0514 (4)	0.0473 (5)	
C4	0.67524 (12)	0.5798 (2)	0.8384 (3)	0.0436 (5)	
C5	0.60947 (12)	0.4866 (2)	0.7692 (3)	0.0385 (5)	
C6	0.58325 (11)	0.38356 (19)	0.9157 (3)	0.0337 (4)	
C7	0.50735 (12)	0.28755 (19)	0.8387 (3)	0.0390 (5)	
C8	0.57289 (12)	0.0497 (2)	0.9043 (4)	0.0417 (5)	
C9	0.57559 (11)	−0.0814 (2)	1.0552 (4)	0.0422 (5)	
H1	0.5656	0.2261	1.2298	0.059*	
H2	0.7224	0.4609	1.3462	0.054*	
H3	0.7650	0.6328	1.0950	0.056*	
H4	0.5819	0.4926	0.6198	0.047*	
H5	0.5090	0.2653	0.6841	0.046*	
H6	0.4562	0.3383	0.8565	0.046*	
H7	0.5580	0.0195	0.7531	0.051*	
H8	0.6272	0.0954	0.9174	0.051*	
H9	0.6173	−0.1479	1.0157	0.051*	
H10	0.5898	−0.0510	1.2065	0.051*	

### Atomic displacement parameters (Å^2^)

**Table:**

	*U*^11^	*U*^22^	*U*^33^	*U*^12^	*U*^13^	*U*^23^
Cl1	0.0679 (4)	0.0428 (3)	0.0828 (5)	−0.0173 (2)	0.0324 (3)	−0.0034 (2)
O1	0.0618 (9)	0.0389 (8)	0.0467 (9)	−0.0050 (6)	−0.0016 (7)	−0.0006 (6)
N1	0.0266 (6)	0.0258 (6)	0.0545 (10)	−0.0007 (5)	0.0077 (6)	−0.0043 (6)
C1	0.0365 (9)	0.0272 (8)	0.0493 (11)	0.0049 (7)	0.0029 (8)	−0.0052 (8)
C2	0.0383 (10)	0.0387 (10)	0.0582 (13)	0.0016 (8)	−0.0081 (9)	−0.0107 (9)
C3	0.0323 (9)	0.0367 (9)	0.0727 (15)	−0.0036 (8)	0.0044 (9)	−0.0146 (10)
C4	0.0363 (9)	0.0299 (9)	0.0670 (14)	−0.0038 (7)	0.0158 (9)	−0.0074 (8)
C5	0.0372 (9)	0.0304 (9)	0.0481 (11)	−0.0024 (7)	0.0057 (8)	−0.0045 (8)
C6	0.0295 (8)	0.0255 (7)	0.0463 (10)	0.0007 (7)	0.0043 (7)	−0.0059 (7)
C7	0.0349 (9)	0.0302 (9)	0.0510 (12)	−0.0037 (7)	0.0002 (8)	−0.0011 (8)
C8	0.0314 (8)	0.0295 (9)	0.0665 (13)	−0.0012 (7)	0.0153 (8)	−0.0067 (8)
C9	0.0286 (8)	0.0293 (8)	0.0689 (13)	−0.0005 (7)	0.0062 (8)	−0.0048 (9)

### Geometric parameters (Å, °)

**Table:**

Cl1—C4	1.750 (2)	C8—C9	1.510 (2)
O1—C1	1.360 (2)	O1—H1	0.832
N1—C7	1.474 (2)	C2—H2	0.950
N1—C8	1.468 (2)	C3—H3	0.950
N1—C9^i^	1.468 (2)	C5—H4	0.950
C1—C2	1.395 (2)	C7—H5	0.950
C1—C6	1.399 (2)	C7—H6	0.950
C2—C3	1.375 (3)	C8—H7	0.950
C3—C4	1.377 (3)	C8—H8	0.950
C4—C5	1.376 (2)	C9—H9	0.950
C5—C6	1.389 (2)	C9—H10	0.950
C6—C7	1.520 (2)		
			
C7—N1—C8	112.58 (15)	C3—C2—H2	120.2
C7—N1—C9^i^	111.96 (14)	C2—C3—H3	120.7
C8—N1—C9^i^	109.43 (14)	C4—C3—H3	120.0
O1—C1—C2	117.87 (17)	C4—C5—H4	119.7
O1—C1—C6	122.46 (16)	C6—C5—H4	119.7
C2—C1—C6	119.67 (18)	N1—C7—H5	108.8
C1—C2—C3	120.75 (19)	N1—C7—H6	108.9
C2—C3—C4	119.27 (18)	C6—C7—H5	108.3
Cl1—C4—C3	119.17 (14)	C6—C7—H6	109.0
Cl1—C4—C5	119.92 (16)	H5—C7—H6	109.5
C3—C4—C5	120.90 (19)	N1—C8—H7	109.3
C4—C5—C6	120.60 (18)	N1—C8—H8	109.5
C1—C6—C5	118.68 (16)	C9—C8—H7	108.6
C1—C6—C7	121.77 (17)	C9—C8—H8	110.1
C5—C6—C7	119.41 (16)	H7—C8—H8	109.5
N1—C7—C6	112.28 (15)	N1^i^—C9—H9	109.3
N1—C8—C9	109.81 (17)	N1^i^—C9—H10	109.5
N1^i^—C9—C8	109.75 (15)	C8—C9—H9	110.4
C1—O1—H1	108.6	C8—C9—H10	108.5
C1—C2—H2	119.0	H9—C9—H10	109.5
			
C7—N1—C8—C9	175.34 (15)	C6—C1—C2—C3	3.6 (3)
C8—N1—C7—C6	−74.1 (2)	C1—C2—C3—C4	−0.6 (3)
C7—N1—C9^i^—C8^i^	−175.02 (17)	C2—C3—C4—Cl1	176.21 (16)
C9^i^—N1—C7—C6	162.11 (16)	C2—C3—C4—C5	−2.3 (3)
C8—N1—C9^i^—C8^i^	59.4 (2)	Cl1—C4—C5—C6	−176.38 (15)
C9^i^—N1—C8—C9	−59.5 (2)	C3—C4—C5—C6	2.1 (3)
O1—C1—C2—C3	−175.83 (18)	C4—C5—C6—C1	0.9 (2)
O1—C1—C6—C5	175.67 (17)	C4—C5—C6—C7	176.70 (17)
O1—C1—C6—C7	0.0 (2)	C1—C6—C7—N1	−27.3 (2)
C2—C1—C6—C5	−3.8 (2)	C5—C6—C7—N1	157.10 (17)
C2—C1—C6—C7	−179.42 (17)	N1—C8—C9—N1^i^	59.7 (2)

Symmetry codes: (i) −*x*+1, −*y*, −*z*+2.

### Hydrogen-bond geometry (Å, °)

**Table:**

*D*—H···*A*	*D*—H	H···*A*	*D*···*A*	*D*—H···*A*
O1—H1···N1	0.83	1.85	2.604 (2)	150
C3—H3···O1^ii^	0.95	2.60	3.547 (2)	175

Symmetry codes: (ii) −*x*+3/2, *y*+1/2, −*z*+5/2.
